# Selective morphological analysis of cerium metal in electrodeposit recovered from molten LiCl-KCl eutectic by radiography and computed tomography

**DOI:** 10.1038/s41598-018-38022-3

**Published:** 2019-02-04

**Authors:** Young Taek Jee, Miran Park, Seungryong Cho, Jong-Il Yun

**Affiliations:** Department of Nuclear and Quantum Engineering, KAIST, 291 Daehak-ro, Yuseong-gu, Daejeon 34141 Republic of Korea

## Abstract

This paper presents, for the first time, a study to analyze the surface morphology of metal extracted from a high temperature molten salt medium in the electrodeposit using x-ray radiography and computed tomography. Widely used methods such as scanning electron microscopy and inductively coupled plasma-optical emission spectrometry/mass spectrometry are destructive and the related processes are often subject to the air condition. The x-ray imaging can provide rich information of the target sample in a non-destructive way without invoking hydrolysis or oxidation of a hygroscopic sample. In this study, the x-ray imaging conditions were optimized as following: tube voltage at 100 kVp and the current exposure time product at 8.8 mAs in our in-house x-ray imaging system. LiCl-KCl and cerium metals used in this work produced substantially distinguishable contrasts in the radiography due to their distinctive attenuation characteristics, and this difference was well quantified in the histograms of brightness. Electrodeposits obtained by chronoamperometry and chronopotentiometry demonstrated a completely different behavior of electrodeposition even at the same applied charge. In particular, computed tomography and volumetric analysis clearly showed the structural and morphological dissimilarity. The structure of cerium metal in the electrodeposit was successfully separated from the chloride salt structure in the CT image by an image segmentation process.

## Introduction

Electrolytic extraction, accompanied by molten salt, is one of the widely used techniques for selective recovery of various target elements. In particular, for extracting rare earth elements, various studies on selection of specific molten salt and/or electrode are actively conducted to enhance the efficiency of extraction^1–5^. In the nuclear industry, pyroprocessing that can recover uranium and transuranic elements in a non-proliferative way, has been proposed as a promising technology for recycling the used nuclear fuel and for reducing radioactive nuclear waste^6^. The most important unit process in the pyroprocessing is electrorefining, during which actinides are dissolved into molten salt and selectively extracted. At an applied potential of −1.4 V (vs. 1 wt% Ag|AgCl), the uranium(III) chloride ion dissolved in molten LiCl-KCl can be reduced to uranium metal^7^. A large amount of electrodeposit would be formed in a metallic form at the cathode since most of the uranium can be recovered by this process. It is reported that the uranium deposit has a dendritic structure^8^, which drastically increases the electroactive surface area of the working electrode. Change of the surface area during the electrorefining process continuously alters the entire electrochemical conditions such as the current density, the electric field strength near the electrode, the potential and current gradient along the diffusion layer, and the exchange current, etc. So far, however, there has been no study on the surface change of the working cathode during the process in molten salt, and only a few studies on the kinetics of the anodic dissolution have been performed^9–11^. Moreover, there is no simulation model for the pyroprocessing that reflects the transient phenomenon of the working cathode. Indeed, all the existing simulations assume that the surface area of the electrode and the related electrochemical conditions remain constant^10,12^.

Destructive analysis is a popular approach to investigating electrodeposits due to the extreme conditions of the pyroprocessing including a temperature of 773 K in an inert atmosphere free of H_2_O or O_2_. However, it is difficult to maintain a salt sample unchanged in such a destructive analysis because of readily occurring reactions such as hydrolysis and/or oxidation in the aerobic environment. The hygroscopic nature of LiCl-KCl leads to a change in the texture of electrodeposits from the solid to a mud-like distorted metal structure. The inherent aerobic properties of electrolytic recovery in high temperature molten salt, therefore, strictly limit the scope of the analysis to either the recovered quantity or the microstructural morphology of a dimension of a few μm and give one-off opportunity for any electrodeposit. Typically, quantitative evaluation of the amount of electrodeposits is often performed by inductively coupled plasma-optical emission spectrometry (ICP-OES) or inductively coupled plasma-mass spectrometry (ICP-MS) to assess the efficiency of recovery. Scanning electron microscopy (SEM), scanning electron microscopy-energy dispersive spectrometry (SEM-EDS), and atomic force microscopy (AFM) are often used for qualitatively analyzing the morphology of the electrodeposit^13–19^. There are three critical limitations in these analysis methods. First, a sample cannot be used for both qualitative and quantitative evaluation at the same time. Second, the electrodeposit also contains an arbitrary amount of the background LiCl-KCl electrolyte in its structure, which may confound quantitative analysis of the recovery efficiency. Finally, the microscopy-based morphology analysis would yield local characteristics of the electrodeposit rather than the entire sample characteristics. Because of these hurdles, fragmentary information can be obtained from a deposit sample in a scattered fashion. In this work, we propose to use x-ray radiography and computed tomography (CT) for selective analysis of morphology of the metallic deposits as an attempt to address the aforementioned limitations of the existing techniques. Exploiting the advantages of the radiography and CT, we compare two electrochemical techniques, chronoamperometry (CA) and chronopotentiometry (CP), that are in common uses. In addition, volumetric analysis by counting the number of effective voxels of the reconstructed deposits is carried out, and their actual amount recovered on the electrode can be calculated by multiplying the density. This technique enables both qualitative analysis on the internal structure of pure metal deposits and quantitative analysis on the actual amount recovered on the electrode at the same time. Cerium(III) was chosen to be the target material in this work, for uranium-based experiment in an academic laboratory setting is difficult and for it is well known to be a good surrogate of uranium^7,20^.

## Results

### Electrochemical system and electrodeposition of cerium(III)

**Figure 1:**
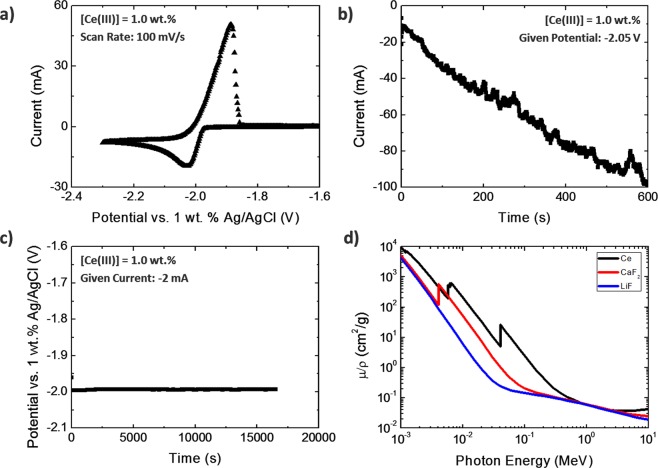
(**a**) Cyclic voltammogram (CV) with a scan rate of 100 mV/s. (**b**) Chronoamperometry (CA) with an applied voltage of −2.05 V for 600 seconds. (**c**) Chronopotentiometry (CP) with an applied current of −2 mA for 16,500 seconds for 1 wt% of cerium(III) in LiCl-KCl molten salt. (**d**) Mass attenuation coefficients of cerium metal and various salts^22^.

Cerium metal was recovered from LiCl-KCl molten salt using two different electrochemical techniques, CA and CP. Prior to electrochemical deposition, a cyclic voltammogram of LiCl-KCl-CeCl_3_ at a scan rate of 100 mV/s was measured to obtain the electrochemical properties of cerium(III), as shown in Fig. 1a. The well-known reduction peak of cerium(III) was observed at −2.02 V^21^. Concentration of cerium(III) in all the experiments was adjusted to be 1.0 ± 0.2 wt%. At this constant concentration, the immersed depth of the electrode was double-checked against the magnitude of the reduction peak. The current of −22 ± 1 mA was reproduced in all the experiments with an immersed electrode depth of 1.0 cm. In order to maintain a stable reduction environment, a constant voltage of −2.05 V was applied across the electrodes in the system during CA. As shown in Fig. 1b, a negative current with its magnitude gradually increasing from 10 mA to 100 mA during 600 seconds of CA process was observed. The linear increase in magnitude of the current is thought to be due to increasing electroactive surface area of the working electrode.

In the CP experiment, a stable potential across the electrodes of −1.98 V was measured during the electrodeposition for 16,500 seconds by applying a constant current of −2 mA as shown in Fig. 1c. In CP, if a current supply overwhelms, cerium(III) ion in the diffusion layer would be fast depleted and the potential may pass the reduction potential of cerium(III) reaching the potential of Li(I) reduction at −2.50 V. Therefore, the stable potential with its value under the reduction peak of Li(I) in Fig. 1c implies that a stable electrodeposition of cerium(III) selectively has occurred. Although a database of the mass attenuation coefficient of LiCl-KCl is not publicly available, it was able to determine the validity of the x-ray analysis of deposits recovered from molten salts by differences of density and composition between the target metal and the co-collected salt medium. The mass attenuation coefficient of cerium metal was compared with that of other similar halogen salts, and its larger mass attenuation coefficient of cerium metal is shown in Fig. 1d ^22^.

### Scanning parameters of the radiography system

**Figure 2:**
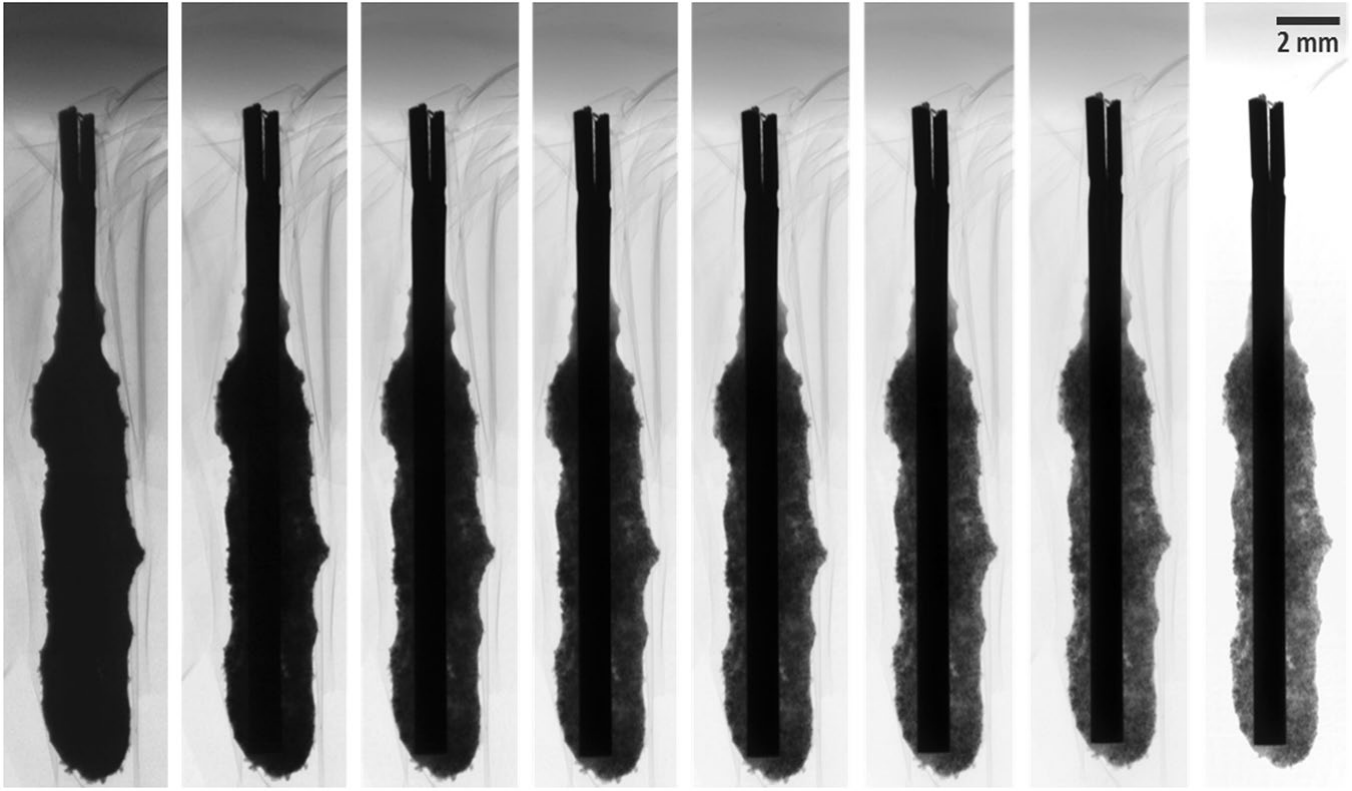
Radiography of a deposited sample obtained by 10,000 second by CP with a constant current of −7 mA at various voltages, 20, 30, 40, 50, 60, 80, 100, and 120 kVp at a constant current-exposure time product of 8.8 mAs (from the left). The darkest rod in each image is the tungsten electrode. Display window is set to be [0, 16384] a.u. (Supplementary Video S1).

The electrodeposit, whose radiographic images are shown in Fig. 2, was made by CP at a constant applied current of −7 mA for 10,000 seconds. The sample was then fixed to a jig with a tight sealing and went through radiographic scans. The tube voltage is directly related to the image contrast, and the tube current is associated with the image noise. When the x-ray tube voltage is high, the image pixel values can saturate and the visibility of low-attenuation objects, as shown in the case of 120 kVp in Fig. 2, may be sacrificed. When low, on the other hand, the high-attenuation structures in the radiographic images may become indistinguishable, as shown in the cases of up to 40 kVp in Fig. 2. The optimum tube voltage was determined to be 100 kVp from a visual inspection of the radiographic images and their display windows in Fig. 2. A higher tube current is desirable since it would produce a low-noise radiographic image. However, as the tube current increases the x-ray focal spot size increases as well. Consequently, the image would become blurred as the size of the focal spot increases^23^. The scanning condition of 100 kVp at 80 μA was used in this work.

### Quantitative analysis of the images

**Figure 3:**
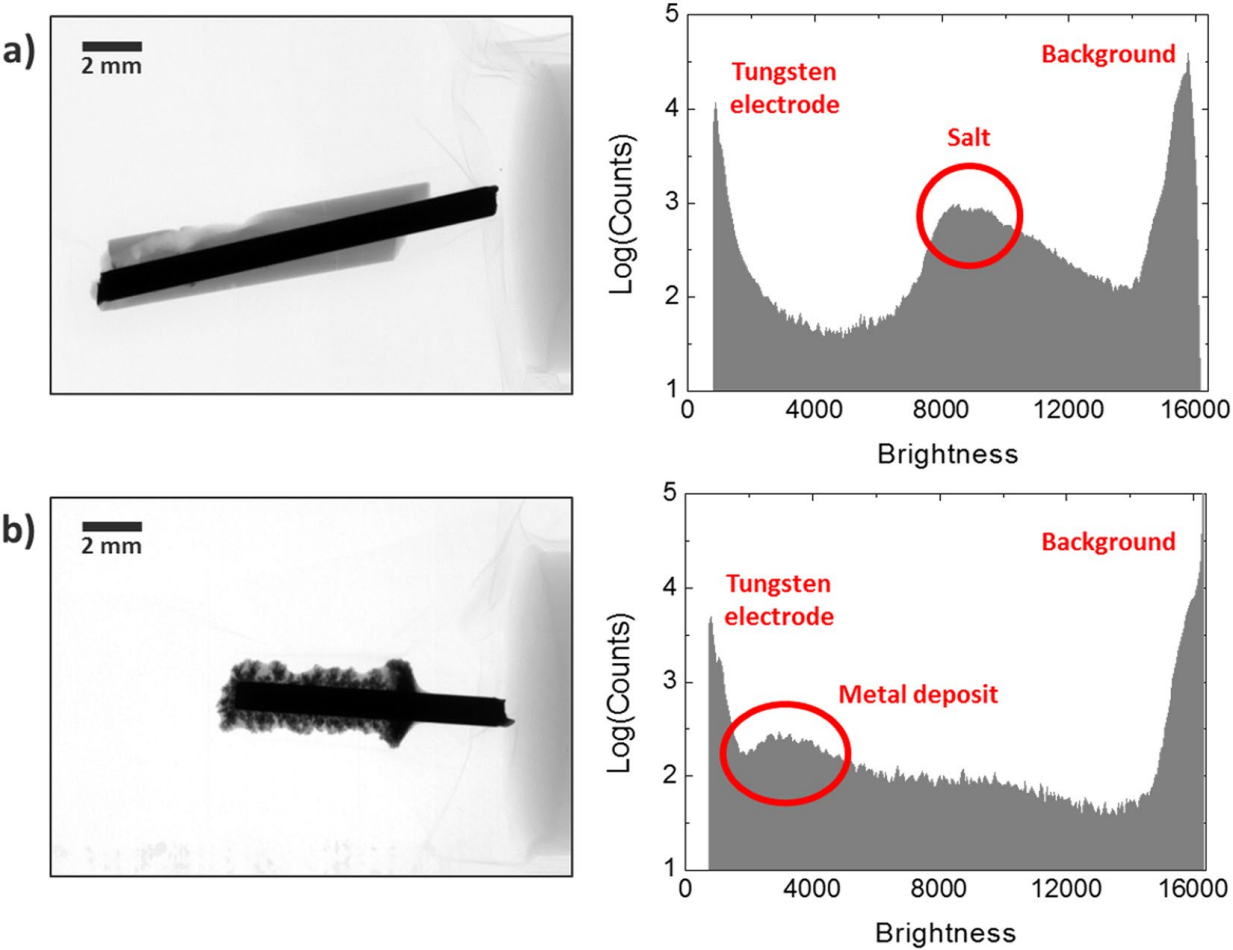
Radiography and gray-scale histograms of (**a**) bulk LiCl-KCl-CeCl_3_ (Supplementary Video S2). (**b**) Recovered cerium metal at the tungsten electrode (Supplementary Video S3).

Samples of bulk LiCl-KCl-CeCl_3_ and densely deposited cerium metal with a small amount of trapped salt were prepared to show the quantitative difference in the attenuation of x-ray. A salt rod, whose radiographic image is shown in Fig. 3a, was prepared by extracting the molten salt with a pyrex tube of which diameter is 2 mm. Electrodeposit, as shown in Fig. 3b, was produced by flowing a constant current of −2 mA for 9 hours. From the radiographic image in Fig. 3b, a mossy morphology of metal (dark gray) surrounded by salt (light gray) can be readily observed, while the other image in Fig. 3a shows salt only. In both histograms, two common peaks were observed: a peak in the dark zone representing the tungsten electrode due to the high attenuation of x-ray and another peak in the bright zone representing the background. Additionally, the histogram in Fig. 3a shows a peak near the brightness of 8,500, while the other histogram in Fig. 3b shows a peak around 3,500 with a smaller count. The scattered histogram from 3,000 to 8,000 is also observable in Fig. 3b and is thought to be related to the inhomogeneity of the deposit formation along the electrode. On the other hand, a sharper peak corresponding to the blank salt is thought to reflect its homogeneity in composition. The broadness of the peak relates to the difference in thickness from the radial distribution of cylindrical samples. Since the thicker the sample, the greater the attenuation, the broadness of the peaks in the histogram can be varied with respect to the penetration depth. The inherent differences in the density and the composition of cerium metal and LiCl-KCl salt, however, give large differences in beam attenuation, which describes the difference in peak position. Comparison between the two histograms, therefore, confirms that a radiography helps to visualize and thus distinguish salt and metal deposits in a non-destructive way.

### Application of radiography and CT: Visualization of metal structure in electrodeposits produced from two different electrochemical techniques

**Figure 4:**
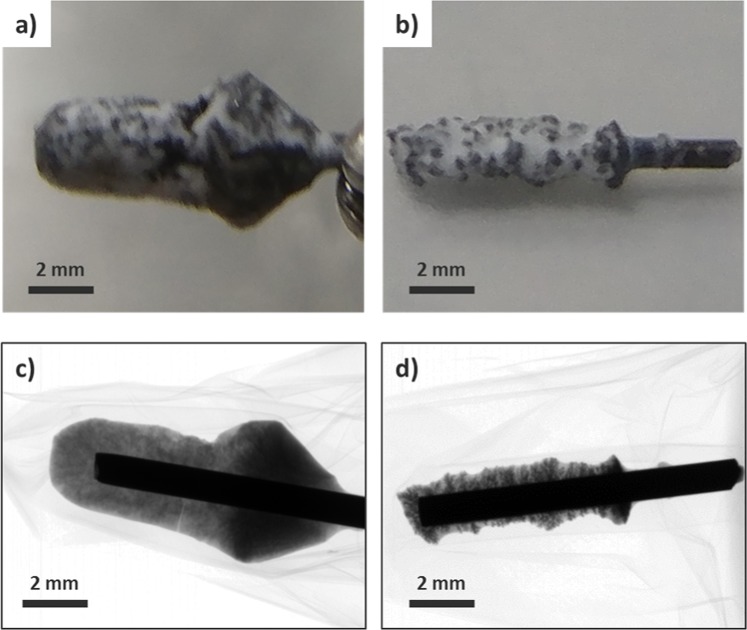
(**a**) Photograph of electrodeposit obtained by CA at −2.05 V for 600 seconds. (**b**) Photograph of electrodeposit obtained by CP at −2 mA for 16,500 seconds. (**c**,**d**) Their radiographic images (rotational view: Supplementary Videos S4 and S5), both 33 coulombs (current (I) × time (s)) of charge flow.

Electrodeposits produced by two different electrochemical techniques showed substantial differences in their size and structure. Figure 4a,b show the pictures of the two different electrodeposits produced by CA and CP with the same total electric charge of 33 coulombs, which corresponds to a deposition of 0.016 g cerium metal. The electrochemical results of both reactions are shown in Fig. 1. CA took 600 seconds at an average current of −55 mA, while CP took 16,500 seconds at a fixed current of −2 mA. As shown in Fig. 4c,d, the radiographic image of the cerium metal obtained by CA formed a cloudy and well-spread structure filled with LiCl-KCl, while that by CP produced an agglomerated mossy structure.

**Figure 5:**
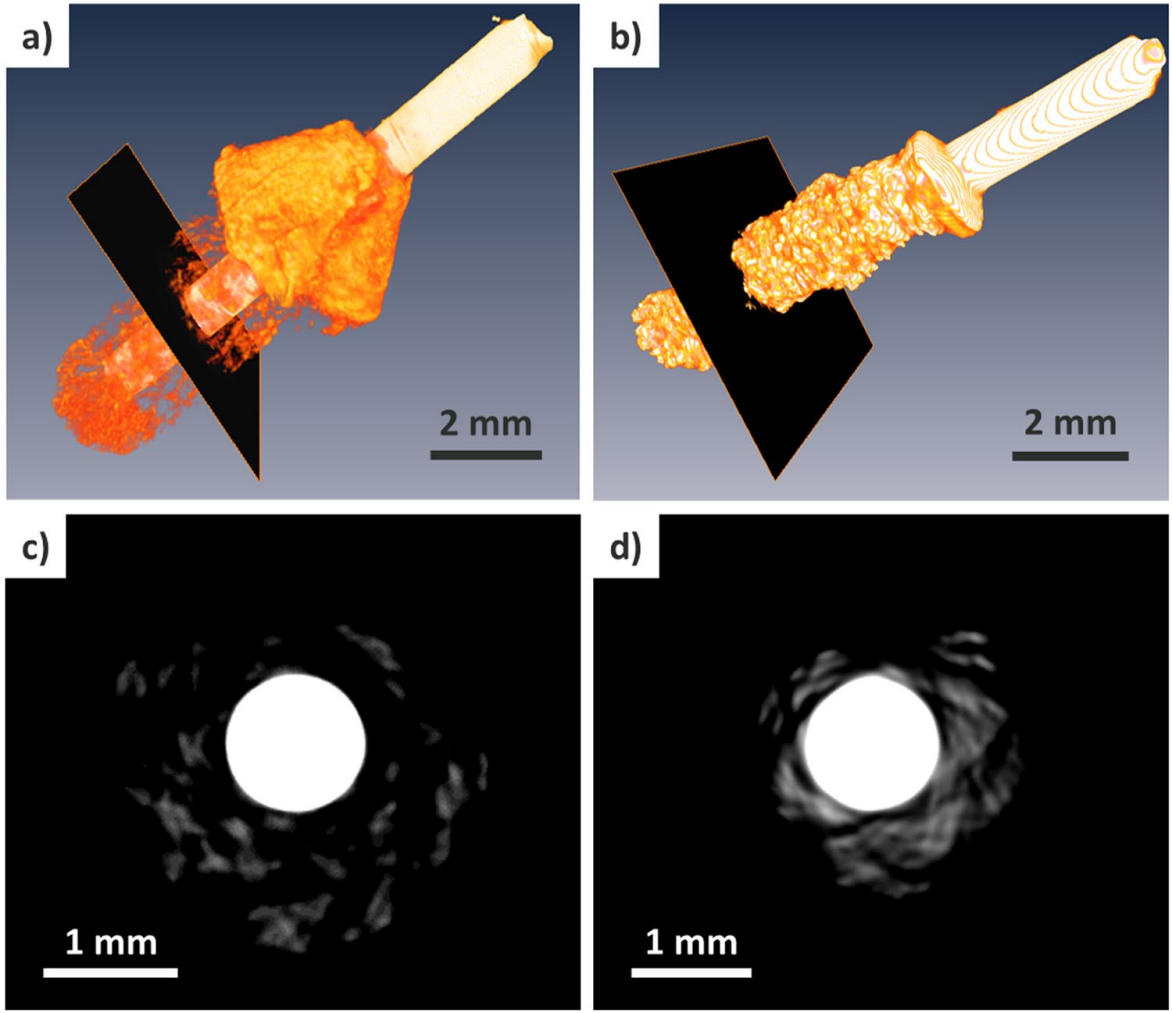
Reconstructed CT images, (**a**,**b**). Slice images of reconstructed electrodeposits, (**c**,**d**), described in Fig. 4.

**Figure 6:**
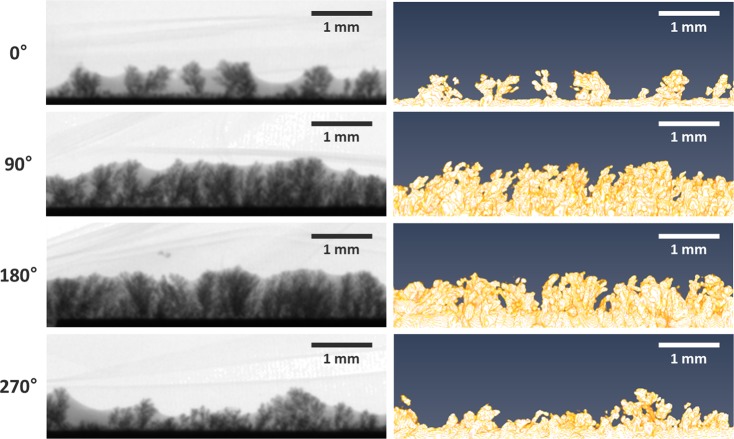
Magnified version of locally agglomerated electrodeposit at the surface of electrode in Fig. 5b. Radiographic images (left) and corresponding reconstructed CT images (right) are visually comparable even at different angular positions: 0°, 90°, 180°, and 270°.

A better visualization of the morphological contrast can be achieved by three-dimensional reconstruction of CT images. By controlling the display window, only the structure of cerium can be visualized. As shown in Fig. 5a,b, CA electrodeposit containing a considerable amount of LiCl-KCl can be visually separable from the cerium structure. Cut-view of the reconstructed images, as shown in Fig. 5c,d, shows the differences in structural morphology and density. The bright central circle corresponds to the tungsten electrode and the remainder of gray structures to the cerium metal. Figure 5d in comparison with Fig. 5c confirms that the cerium metal is more densely deposited at the electrode by CP than CA. There exist concerns about metal artifacts in the CT images particularly due to photon starvation and scattering across the electrode. Theoretically, 1 mm-thick tungsten requires the x-ray energy of about 150 keV or higher for acquiring transmission data that is photon starvation free^24^. In the current CT scanning condition that uses the tube voltage of 100 kVp, therefore, metal artifacts can occur. This will effectively result in a limited-angle tomography where only particular part of the whole rotational scan data would practically contribute to the reconstruction of a given region of the metal deposits surrounding the tungsten rod. There are a host of metal artifact reduction methods in CT imaging with varying degrees of artifact reduction. The reduction methods primarily aim at reducing the artifacts emanating from the metal in the soft-tissue images. Unfortunately, those methods would not be directly applicable in this problem since the imaged object is mostly composed of metals and the region-of-interest is also metal deposit. Interestingly, even without using such reduction methods, we have noticed that the reconstructed 3D structural information of the metal deposits, which is the primary imaging target or the region-of-interest in CT scanning in this study, is quite intact in comparison with the corresponding radiographic images, as shown in Fig. 6.

### Conventional analyses for reference

Experiments to further assess the distinct behavior of electrodeposition based on the two electrochemical techniques were additionally performed. The products have been quantitatively analyzed using mass balance and ICP-OES. The results are summarized in Table 1.

**Table 1:** Results of the mass difference of electrode before and after deposit dissolution with 3% nitric acid solution and the mass of cerium metal deposited in triplicate experiments.

	Set 1 (33 coulombs)		Set 2 (33 coulombs)		Set 3 (30 coulombs)	
	CA	CP	CA	CP	CA	CP
Expected amount (mg) (in wt%)	16.0 (0.0622)	16.0 (0.0622)	16.0 (0.0622)	16.0 (0.0622)	14.5 (0.0565)	14.5 (0.0565)
Δ Mass of electrode (mg)	151 ± 2	31 ± 2	143 ± 2	48 ± 2	93 ± 2	26 ± 2
Ce metal by ICP-OES (mg)	15.3 ± 0.3	6.4 ± 0.4	15.9 ± 0.8	5.9 ± 0.2	12.1 ± 0.8	5.5 ± 0.9
Calculated mass of LiCl-KCl (mg)	136 ± 3	25 ± 3	127 ± 3	42 ± 3	81 ± 3	20 ± 3
Collection efficiency (%)	95 ± 3	39 ± 3	99 ± 5	37 ± 2	83 ± 6	38 ± 7
Δ Bulk (Ce(III)) concentration (wt%)	−0.061 ± 0.004	−0.060 ± 0.003	−0.059 ± 0.002	−0.059 ± 0.002	−0.055 ± 0.002	−0.054 ± 0.003

In three electrochemical sets (CA and CP), similar amount of charge was provided for each electrodeposition at 33 coulombs for set 1 and 2 and 30 coulombs for set 3. Expected amount of electrodeposit in terms of both mass (mg) and concentration (wt%) calculated based on transferred charge is addressed in the first row of Table 1. The mass of electrode was measured before and after dissolving the electrodeposit in 3% nitric acid. The dissolved deposit was analyzed with ICP-OES for the quantitative analysis of cerium, and the mass of LiCl-KCl trapped on the electrodeposit was calculated by subtracting the mass of cerium from the mass difference of electrode. The collected deposit on the electrode from CA experiments showed four to five times larger mass difference than those from CP experiments. The amount of LiCl-KCl trapped in the electrodeposit of CA, therefore, was measured to be larger than that in the electrodeposit of CP. This result clearly explains the larger size of electrodeposit by CA despite of the same supply of the electric charge. The collection efficiency by CA was 91 ± 8%, while that by CP reached 38 ± 7% in average. However, the difference in the concentration of cerium(III), measured by ICP-OES, in the bulk before and after each individual electrodeposition showed a consistency with the theoretical amount calculated with transferred charge. Each set, therefore, substantiated that the expected amount of charge was well transferred and that the expected amount of cerium(III) was successfully converted to cerium(0). Discrepant results between the mass of cerium collected and the change in bulk concentration in the CP experiment are due to the fact that there exists a loss of electrodeposit by physical detachment from the electrode under the CP environment.

## Discussion

Cerium metal has a larger mass attenuation coefficient than the analogous salts (LiF or CaF_2_), as shown in Fig. 1d, and this comparison is also assured in the LiCl-KCl case. This is largely related to the density and Z number of materials. The density of cerium metal (Z = 58) is 6.77 g/cm^3^, whereas LiCl-KCl (Z = 3, 17, 19) is 2.02 g/cm^3^ at room temperature. Therefore, x-ray imaging can provide an efficient morphological analysis of the products of such an electrodeposition.

**Figure 7:**
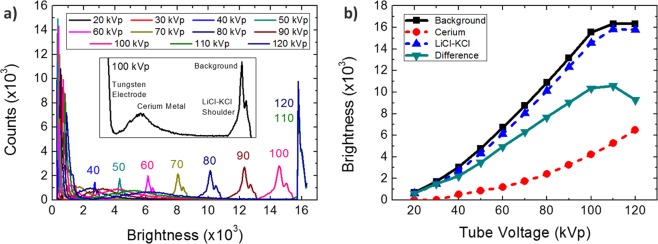
Analysis of the radiographic images shown in Fig. 2: (**a**) Brightness histograms at various tube voltages from 20 kVp to 120 kVp with a constant current-exposure time product of 8.8 mAs are shown. (**b**) Representative brightness of cerium (red circle, dotted) and LiCl-KCl (blue triangle, dotted), the difference in brightness between them (turquoise inverted triangle, solid), and the background (black square, solid) at various tube voltages are plotted.

**Figure 8:**
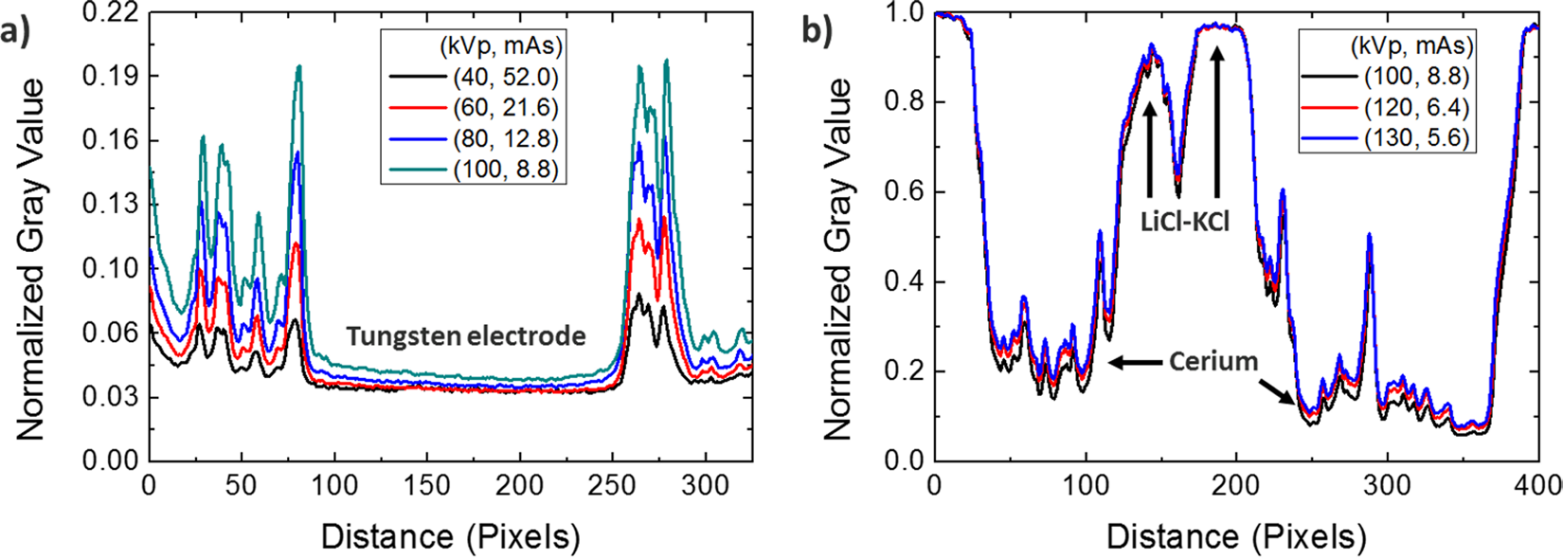
(**a**) Line profiles across the electrodeposits show the increasing contrast of cerium metal with respect to the tube voltage. (**b**) Line profiles across both cerium metal and LiCl-KCl show that the radiographic data of LiCl-KCl tends to stay constant at the higher tube voltage than 100 kVp.

The optimization of the x-ray tube voltage was based on the histogram analysis as summarized in Fig. 7. Figure 7a shows the histograms acquired at various tube voltages. Insert of Fig. 7a is labeled with the corresponding material to each peak. Compared to Fig. 3a, the histogram does not show a clear peak of LiCl-KCl, since the sample used in Fig. 7a is a mixture of cerium metal and trapped LiCl-KCl while the sample in Fig. 3a was a pure bulk. The cerium and LiCl-KCl peaks are indistinguishable at 20 and 30 kVp. The brightness of each structure becomes separable at 40 kVp, and the contrast between the metal and the salt increases up to 100 kVp, as can be seen in Fig. 7b. The contrast tends to decrease at higher voltages than 100 kVp primarily due to detector saturation. The LiCl-KCl peak has also been missed. Seeking the optimum tube voltage, we wanted to remove the detector saturation problem. We used varying current-exposure time products for the tested tube voltages securing no detector saturation. The contrast between the tungsten electrode and the cerium metal was strongly enhanced as the tube voltage increases up to 100 kVp, as shown in Fig. 8a. The contrast of the salt with respect to the background at higher voltages tends to stay relatively constant, as shown in Fig. 8b. Therefore, the contrast between the metal and the salt would slightly decrease at higher voltages than 100 kVp. In summary, it was thus confirmed that the experimental conditions of 100 kVp and 8.8 mAs are appropriate for the analysis of electrodeposits in terms of the clear contrast between the tungsten electrode and the cerium metal, the contrast between the cerium metal and the trapped LiCl-KCl salt, and the visualization of the LiCl-KCl structure along the electrodeposits.

Use of two different ways of electrodeposition, CP and CA, has been widely investigated with varying focuses on the properties of products^13,25–27^. CA is known to yield an efficient extraction in a relatively short time with a guaranteed reduction condition by applying a more negative potential for electrodeposition. An increment of current flow during the course of CA would produce a quadratic rise in charge transfer due to the surface enlargement. On the other hand, CP provides a constant current forming a milder electrochemical condition in terms of current density as electroactive surface area increases. Therefore, compared to CA, CP allows a more precise calculation of the charge flow and enables a reasonable prediction of the amount of electrodeposit^28^. In this work, the electrodeposition processes by CA and CP have been revisited by use of x-ray imaging. Transferring a given amount of charge, e.g. 33 coulombs, during 600 seconds or during 16,500 seconds would render completely different electrochemical conditions. CA at a higher charge flow rate, which corresponds to the case of 600 seconds transfer, would result in an enlarged surface area and an intensified current flow in return. Because of a continuous escalation of the induced current signal, the electrodeposit hardly agglomerates in a localized structure but forms a widespread cloud-like structure allowing the molten salt to fill up the void area. On the other hand, CP, which maintains a relatively lower charge flow rate for 16,500 seconds at −2 mA to transfer 33 coulombs, provides a milder electrochemical condition than CA. In this case, the increase of the surface area during the electrodeposition gradually reduces the current density along the electrode. This is due to a stable, well-localized, and conductive electrodeposition of LiCl-KCl mostly on the surface rather than in the internal structure without forming voids. The structural difference can also be analyzed with the volumetric calculation using CT images. By setting the threshold in gray level for cerium metal or total deposit including LiCl-KCl, each volume can be determined by counting the segmented voxels. Similarly, the volume allocated for trapped LiCl-KCl can be calculated by subtracting the volume of cerium from that of the total deposit. The voxel size was 20 × 20 × 20 μm^3^ in the reconstruction, and the calculated results are listed in Table 2. The volume of the entire deposit showed a marked difference due to different amounts of LiCl-KCl in the structure of deposit. Based on the results of volumetric analysis and ICP-OES, it was concluded that the reconstructed image well reflects not only the structure and morphology but also the quantitative analysis of electrodeposit.

**Table 2:** Volumetric analysis for CT images of electrodeposits shown in Fig. 5 by counting the number of voxels (voxel size: 20 × 20 × 20 μm^3^).

Technique	Target	Voxel Counts (#)	Volume (mm^3^)
CA	Cerium	338,439	2.7
	Electrodeposit	8,981,720	71.8
	LiCl-KCl	8,643,281	69.1
CP	Cerium	163,854	1.3
	Electrodeposit	2,109,640	16.9
	LiCl-KCl	1,945,786	15.6

We would like to discuss different collection efficiencies of the two methods in electrodeposition. As summarized in Table 1, the concentration of cerium(III) in the bulk was decreased by an expected amount. However, the collection efficiency of the CP method was substantially lower than that of CA. This is due to the physical detachment of cerium from the electrode that can occur more often in CP than in CA because of the weaker negative potential in CP. The potential of the system measured in CP at −2 mA was −1.98 V while that in CA was −2.05 V.

## Conclusion

X-ray radiography and computed tomography have been successfully applied to a morphological analysis of electrodeposition produced by electrolytic reduction in a molten chloride salt environment for the first time to the best of the authors’ knowledge. X-ray imaging enabled a macroscopic and qualitative analysis of the electrodeposit, which might not be available due to its microscopic nature in SEM, TEM, or FIB-SEM analyses. The reconstructed images provided anatomical structure of the electrodeposits in a three-dimensional form. Morphologic differences between the electrodeposits generated by CA and CP have thus been visually demonstrated, and the size dependency of electrodeposits on the physical trapping of LiCl-KCl, was clarified. It was confirmed that the electrodeposit by CA contains much LiCl-KCl within the sparse metal structure, while that by CP contains LiCl-KCl at the surface of densely packed cerium metal. It was possible to perform a surface or morphology analysis with the salt being separated from the target metal. Additionally, the structural difference was quantitatively verified by the volumetric calculation from CT images. The proposed non-destructive analysis would not only be utilized in a transient electrode analysis of pyroprocessing but also be extended to other morphological analyses in a high temperature medium susceptible to an aerobic environment.

## Methods

### Electrochemical experiments in molten salt system

All preparations and experiments were performed in a glove box filled with high purity argon gas (99.9999%). The glove box system was controlled to keep the concentrations of H_2_O and O_2_ below 5 ppm under a constant circulation of argon gas for an inert environment. The eutectic was prepared by mixing 44 wt% LiCl and 56 wt% KCl (99%, anhydrous, Sigma-Aldrich), and cerium(III) in a chloride form of CeCl_3_ (99.99%, anhydrous, Sigma-Aldrich) was added to a system as a target lanthanide. The sample was placed in a high temperature furnace which maintains 773 ± 3 K stably. Two tungsten electrodes were used each of which plays a working electrode and a counter electrode respectively, and a silver wire immersed in 1 wt% LiCl-KCl-AgCl functioned as a reference electrode. This three-electrode-system was installed in an alumina crucible containing LiCl-KCl eutectic salt. All electrochemical experiments were performed with a potentiostat (Autolab, PGSTAT302N) at a constant concentration of 1 wt% cerium(III). Cyclic voltammetry (CV) was obtained in every single trial to check the stability of the chloride eutectic system. Chronoamperometry (CA) and chronopotentiometry (CP) were utilized as two different electrochemical methods for electrodeposition. All potentials in this work are expressed in values versus the reference electrode with 1 wt% Ag|AgCl.

### Experimental conditions for computed tomography (CT)

The working electrode after the electrochemical deposition was carefully cut with a cutting nipper and mounted on a jig for radiography. The sample was tightly sealed with multiple tape layers inside the inert atmospheric glove box and then brought to radiography for preventing hydrolysis or oxidation during the analysis. Please note that the tape layers are way more radio-transparent than the sample and that their contrast would be outweighed by the sample contrast in radiography. We used a bench-top CT scanner, which consists of the microfocus x-ray source (Hamamatsu L10801) and the CMOS flat panel x-ray detector (Dexela 1207) with a pixel size of 74.8 μm. The tube voltage was varied at a constant tube current-exposure time product of 8.8 mAs. A total of 250 radiographic images were obtained at 1.44° angular interval by rotating the sample, and each projection data was prepared by averaging 10 projections to enhance image quality. The distances from the x-ray source to the detector and to the center of rotation were 190 mm and 50 mm, respectively. Image reconstructions were performed by an analytic cone-beam CT imaging algorithm^29^ accelerated by graphic processing unit (GPU) with a voxel size of 20 × 20 × 20 μm^3^. An inert environment during radiography was confirmed by comparing the first and the last image taken after one complete rotation, since a distinguishable radiographic distortion may occur when the salt sample undergoes hydrolysis or oxidation.

## Supplementary information

Supplementary Video S1

Supplementary Video S2

Supplementary Video S3

Supplementary Video S4

Supplementary Video S5
